# Regional variations in the rural-urban fertility gradient in the global South

**DOI:** 10.1371/journal.pone.0219624

**Published:** 2019-07-19

**Authors:** Mathias Lerch

**Affiliations:** Max Planck Institute for Demographic Research, Rostock, Germany; Universidade Federal de Minas Gerais, BRAZIL

## Abstract

The study of regional trends in the rural-urban fertility gradient helps us to understand the pace of completion of the fertility transition and the geography of urban growth in the global South. We question whether the hypothesized inverted U-shaped evolution in rural excess fertility is confirmed in four developing regions, and investigate the underlying fertility dynamics by place of residence. Using multiple surveys for 60 developing countries, we analyze long-term rural and urban trends in cohort fertility. The regional comparison is controlled for the international heterogeneity in the stages attained in the fertility transition and the context of urbanization. We found a clearly inverted U-shaped trend in the rural-urban fertility gradient in Latin America, the Middle East and Northern Africa. In Asia, rural excess fertility remained limited. In sub-Saharan Africa it increased monotonically until the most recent cohorts. These differences stem from variations in the urban-to-rural diffusion of the onset of fertility transition and, in sub-Saharan Africa, from a slower pace of decline in rural areas.

## Introduction

Starting in the 1960s, the fertility transition was almost complete in Asia and Latin America by 2010–15. However, women in sub-Saharan Africa were still having more than five children on average [1, 2]. A better understanding of these varying pathways towards low fertility can be gained by focusing on fertility differences within populations over time. The comparison of trends by type of residence location is relevant not only because the contexts of reproductive decision making are different for rural and urban inhabitants. The two sub-groups also represent almost equal shares of the population of developing countries since 2015 [3]. The study of regional differentials in the urban-rural fertility gradient over time therefore helps to conceptualise future fertility developments and the geography of urban growth in the global South.

The transformation from a predominantly rural to a predominantly urban society affects the cultural and socioeconomic environment of human reproduction. On the one hand, the earlier and faster pace of structural changes in urban societies leads to lower fertility compared to that of rural populations [4, 5]. The financial and opportunity costs of childbearing increase with the monetarization of society, the expansion of higher level education, and the new avenues for socioeconomic mobility opened up by the development of industrial and service employment. The delivery of modern methods of family planning is also more efficient in spatially concentrated populations. Desires to have fewer children can thus be more effectively implemented. On the other hand, cultural differences also matter for the diffusion of the small family ideal [6–8]. In an urban society, cultural change is particularly fast because of more intense social interaction when compared to a dispersed rural population. The interconnectedness of the developing regions’ urban societies with countries that are more advanced in the fertility transition hastens the onset of behavioral changes. Individualization and cultural diversification in dense urban populations further accelerate the pace of innovation diffusion within cities [9, 10].

According to spatial diffusion theory, the structural and ideational changes in society that motivate lower fertility spread from cities into peripheral areas during the process of regional development and integration [11, 12]. A recent longitudinal re-appraisal of urban and rural fertility trends has confirmed this model [13]: on average in developing countries, the rural-urban gradient followed an inverted U-shaped evolution over time, as suggested by earlier cross-sectional studies [14, 15]. Rural excess fertility increased due to the earlier onset of the fertility transition in urban areas. When the decline also started in rural areas, the fertility gradient by place of residence began to shrink, as the early pace of the rural decline was more sustained when compared to that in the more advanced transitional stage reached in urban areas [13]. Yet the trajectories of decline (once it started) were similar over the course of the urban and rural transitions. This challenges the explanation of lower urban fertility as being driven by faster underlying structural changes in cities, compared to those in rural areas [4]. The evolution of the rural-urban fertility ratio over time stems mainly from the urban-to-rural diffusion of the onset of structural and ideological changes (rather than from their differential paces in the two sectors).

Recent fertility levels are higher in rural than in urban areas in all developing regions [16, 17]. The difference is particularly large in sub-Saharan Africa (SSA), where the rural populations of several countries have not yet experienced any decline [18–20]. The SSA fertility transition not only started later than in other developing regions, but has also progressed at a slower pace so far. This has been related to lower levels of socioeconomic development at the transition onset, a slower pace of subsequent growth, and a pronatalist culture [21, 22]. Yet it remains unclear whether this SSA exceptionalism characterizes both the urban and the rural populations, or only the latter. The slow fertility decline may also be due to a more recent onset of the same inverted U-shaped evolution in the rural-urban fertility gradient observed elsewhere, in combination with a comparatively lower level of urbanization [13]. More generally, we lack a regional comparison of the evolution in the fertility gradient by type of residence. Although cross-sectional assessments have not found patterned regularities in any developing region [17], we provide a longitudinal comparison that accounts for the differences in the onset of fertility transition. This may help in understanding regional differences and conceptualising future fertility developments.

We question whether the inverted U-shaped evolution in rural excess fertility is confirmed in Asia, Latin America and the Caribbean (LACarr), the Middle East and Northern Africa (MENA), and sub-Saharan Africa (SSA). In the next section, we lay out the expectations about the regional differences, given the variations in the level of urbanization, the patterns of change in reproductive behaviors and the urban-to-rural diffusion of modern family planning. We then introduce the data and the methods used to estimate fertility trends over 80 birth cohorts in 60 countries. In the empirical section, we describe the regional trends in the rural-urban fertility ratio and the underlying dynamics by place of residence, while controlling for the international heterogeneity in the demographic and urbanization context. Finally, we summarize the results and discuss their implications for future fertility developments and the geography of urban growth in the global South.

## Regional demographic context and expected rural-urban fertility gradients

Since 1950, the percentage of the population living in urban areas has more than doubled in each of the four developing regions, but there are substantial differences in the levels. In 2010, 79% of the population in LACarr lived in urban areas, 60% in MENA, 43% in Asia (excluding Central Asia), and 36% in SSA ([3]; population-weighted averages based on country-specific results were calculated, excluding Central Asian countries). These differences in the level of urbanization do not correspond to the paces of fertility decline. In LACarr, where more than half of the population has lived in cities since the early 1960s, the total fertility rate (TFR) dropped from 5.9 in 1950 to 2.2 in 2010. The fertility decline was only slightly less marked in the MENA (from 6.6 to 3.1), where the 50%-bar of inhabitants living in urban areas was not reached before the mid-1980s. Despite the low share of the Asian population living in urban areas, its fertility decline was the fastest of all regions (from 6 to 2.2 children). Even though SSA is characterized by a similarly low level of urbanization, this region experienced the slowest decline–from 6.6. to 5.3 children per woman in 2010 [2, 21]. To better understand this regional diversity in the relationship between the national processes of urbanization and the fertility transition, we aim to compare the trends in the rural and urban areas over time.

The evolution of the rural-urban fertility ratio is determined by the pace of three constituent changes: the initial drop in urban fertility, the time lag between the onsets of the urban and rural transitions, and the trajectories of fertility decline in each type of residence location once it has started [13]. These dynamics are likely to vary by region because of differences in the patterns of fertility decline, in the diffusion of modern contraceptive use, and in the levels of urbanization.

The changes in childbearing behaviors varied across developing regions. The fast fertility transitions in LACarr and in Asia have been driven mainly by family limitation, a process in which women stop childbearing after they have reached their target family size [23–27]. By contrast, the spacing and postponement of births of all orders dominated the slower fertility decline in SSA [28–32]. In the early stages of the transition, the idea of a target family size, and the need to stop having children once this target has been achieved, was not prevalent [33]. Birth limitation emerged only recently in vanguard countries of the region, and especially in urban areas [34, 35].

The four developing regions also differ in terms of the diffusion of family planning within countries. In LACarr, the use of modern means of contraception spread at a very fast pace since the early 1960s, especially in cities through an efficient system of delivery in public and private clinics [36]. But the process lagged behind in remote rural areas of the region because of problems of access. These obstacles have been progressively relieved by community-based distribution and social marketing campaigns starting in the 1970s [37, 38]–although some countries which were more advanced in the transition adopted a “laissez-faire” policy. In 2010, LACarr had the highest prevalence rate of modern contraceptives among developing countries (73%; [39]), with small differentials by type of residence location [16].

Starting also in the 1960s, the diffusion of new methods of birth control was particularly fast and sustained in all countries of East and South-East Asia, while Southern Asia lagged behind. Unlike in the case of LACarr, where couples adopted modern family planning on a voluntary basis, there is a legacy of authoritarian population policies in Asia. In the 1970s and 1980s, governments imposed the small family ideal by enforcing the use of modern birth limitation methods even in remote rural areas (such as through forced sterilization; [40, 41]). In 2010, the prevalence of modern contraceptives was 55% and 62% in Southern and South-Eastern Asia, respectively, with the Eastern sub-region heading the ranking [39]. Contraceptive prevalences are similar by type of residence location, except in Southern Asia where the rural level lags behind the urban trend [16].

In MENA, the progress in the diffusion of modern contraceptives was slower and concentrated in big cities. In rural areas, women used these methods primarily to space (rather than to limit) their births [42]. This implies that their impact on fertility decline was less pronounced than in LACarr and Asia. Yet in 2010, the prevalence of modern contraceptives was 54% in MENA [39], with negligible differences by type of residence location [16].

Unlike the steady decrease in unwanted fertility in these three developing regions, the rate has stayed constant over the last 20 years in SSA [43]. There, the level of unmet need for contraception is consistently the highest in worldwide comparison. Only 25% of women in SSA are using modern means of birth control in 2000–15, even though there is a slow upward trend in all countries [44]. In many countries of the region the prevalence rate has not yet crossed the 33%-bar in urban areas, with large urban-rural differentials (up to 27% percentage points; [16]).

This contextual evidence motivates a number of hypotheses about the regional differentials in the evolution of the rural-urban fertility ratio over time. Given the fast diffusion of a stopping pattern of childbearing in urban LACarr, we would expect an early and sharp increase in rural excess fertility. The significant time-lag in the urban-to-rural diffusion of modern contraceptives should have led to a high peak in the fertility gradient by residence. As soon as family planning services became accessible in remote areas, the rural-urban fertility gradient should have started to decrease at a fast pace. The dominance of cities in the region’s population geography may accelerate the diffusion of the new behaviors in rural areas. A similar pattern can be expected for MENA, although rural (excess) fertility may have declined at a slower pace because of a slower spatial diffusion of behavioral changes in a society which was predominantly rural until the 1980s.

The expectations for Asia are competing. On the one hand, the lower level of urbanization implies a slow diffusion of new reproductive behaviors to rural areas. The peak in the rural-urban fertility gradient may thus be high. On the other hand, governments’ commitment to restraining people’s freedom to have large families, and to spreading the use of modern contraceptives in rural areas early on in the fertility transition, may have put a ceiling on rural excess fertility.

In SSA, the early decline in urban fertility may have been slower, when compared to other regions, because it was predominantly driven by birth spacing. This should have led to a slower increase in the rural-urban fertility gradient. However, this rise is likely to be more prolonged because of the important time lag in the urban-to-rural diffusion of modern contraceptives. This also reflects the minority status of the urban population from which behavioral innovation spreads. The subsequent acceleration of the urban fertility decline (due to the diffusion of birth limiting behaviors) should have led to a continuously widening rural-urban fertility gap, as rural fertility decline is likely to remain slow (due to the dominance of birth spacing).

## Analytical strategy

To uncover the regional differences in the trajectories of the urban-rural fertility gradient, we adopt an approach that controls for three major confounding factors in international comparisons [13]. First, national differences in terms of the demographic context are eliminated by studying fertility trends and gradients over the course of the country-specific fertility transitions (rather than over calendar years). In other words, we compare fertility trends at similar stages of the transition. Second, we use a regression-based approach to estimate regional average fertility trends in order to control for the country heterogeneity in the classification of urban and rural populations (see below).

Third, we focus on cohort fertility. Rural and urban trends in period fertility are flawed, respectively, by temporary peaks and troughs of rural-to-urban migration–especially when migratory and fertility decisions are interlinked [45]. Rural women tend to postpone births in order to facilitate mobility towards cities, and recuperate fertility at the destination. This temporarily inflates the urban levels of fertility and deflates the rural levels. Alternatively, women may rush into marriage and have their first birth before rejoining a partner who has out-migrated. In this case, fertility immediately after arrival would be temporarily low. The conventional period TFR only measures the intensity of childbearing before migration in rural areas, and after the move in urban areas. As recent rural-to-urban migrants account for up to 32% of the urban women of childbearing ages in developing countries [17], the period measures of fertility are distorted by the changes in the timing of family formation over the life course, as induced by migration (in a large number of surveys we lack the data to control for this tempo effect of migration). Cohort measures are not affected by this bias. They are also preferred for the analysis of long term trends, as they smooth out short-term variations in fertility which are related to temporary circumstances women face over their life course.

### Data

We used individual-level data on the number of children ever born (i.e. parity) and birth histories of women aged 30 to 49, drawn from 278 World Fertility Surveys (WFS) and Demographic and Health Surveys (DHS). In order to extend the observation windows into early or late stages of the fertility transition in a number of countries, we also relied on 18 Multiple Indicator Cluster Surveys (MICS; organized by the United Nations Children's Fund) and Integrated Public Use Samples of the earliest or most recent population censuses (IPUMS; [46]). To increase the geographic and temporal scope of the study, we also used data from surveys that excluded never-married women. We measured cohort fertility at age 30 and above, at which the overwhelming majority of women had been married in the regions of interest. Therefore, the exclusion of never-married women in a number of surveys does not significantly affect the estimates (see S1 File); period measures, by contrast, would be strongly biased. We did not consider developing countries from the former Soviet Union and small island states because of the importance of international migration, which cannot be controlled for with the data.

This analysis covers 60 developing countries (see S1 File), in which one to twelve surveys/censuses have been carried out. For the MICS and the IPUMS census data that only provide information on parity (but including for women aged 50 to 64), we used the own children method to reconstruct women’s recent birth histories [47]. Our estimation series cover the cohorts born between 1896 and 1982. The average country contributes with 41 birth cohorts, with a minimum of 15 cohorts and a maximum of 80 cohorts.

**Fig 1:**
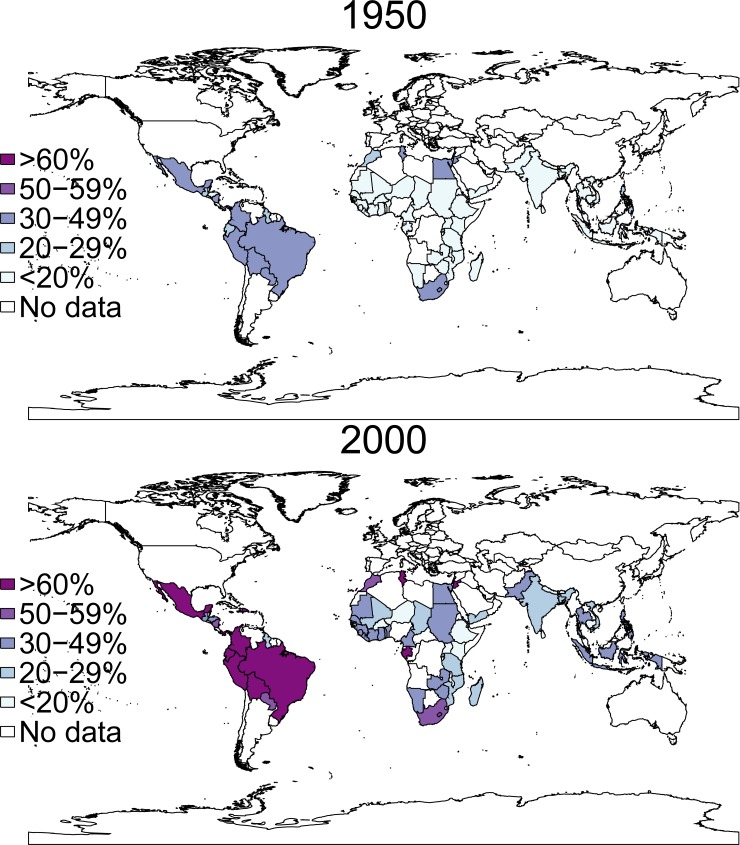
Sample of countries by level of urbanization of the population (in %), 1950 and 2000. Sources: United Nations 2017.

Although SSA countries are overrepresented in the sample due to the greater availability of survey data, regional differences in the urbanization phenomenon are well reflected. The median percentages of the national populations living in urban areas increased from 6% in 1950 to 31% in 2000 among the SSA countries covered in this study, from 13 to 26% among the Asian, from 32 to 53% among the MENA, and from 33 to 61% in the LACarr countries (see Fig 1 and S1 File). The most urbanized and economically developed countries in East Asia and the Southern cone of LACarr are not covered by this analysis (i.e. South Korea, China, Argentina, Chile, Uruguay) because of the absence of standardized survey data.

Given the frequent absence of a clear definition of urban and rural type of residence location in the WFS and DHS, we assume that official definitions are relied upon (as in censuses). These national definitions may change over the years, and the city boundaries move out into formerly rural areas as the population sprawls. However, we can rule out the possibility of major analytical biases due to this rural-to-urban reclassification of populations (or vice-versa), as the fertility trends are cross-validated by successive surveys/census-samples (see S1 File).

International differences in the definition of urban areas pose bigger challenges for comparative research. In some countries, the non-inclusion of slum settlements in officially designated urban areas leads to an over-estimation of the extent of the urban fertility decline because the most fertile urban residents are not considered [48]. In other countries, urban areas include populations with agriculturally-based livelihoods located on the cities’ outskirts [49]. In this case, urban fertility decline would be underestimated. To increase the comparability of the results, we focus on regional average within-country fertility trends in urban and rural areas that are controlled for this (unobserved) intra-regional heterogeneity in the urban/rural definition of space (see below). The regional comparison may still be confounded when different types of urban/rural definitions are more prevalent in different world regions. In SSA, the use of administrative criteria or some low population threshold (between 2,000 and 5,000 residents) tends to include agricultural communities [49]. In Asia, the functional definitions of urban areas (i.e. based on population density, socioeconomic structure, and the availability of urban services) tend to more adequately identify populations living in urbanized living environments [50]. In Latin America, a mix of criteria is used with a majority of countries also relying on administrative criteria and low population thresholds [50]. As we are unable to account for these regional differences in the definitions, the results must be taken with caution.

### Method

We estimated urban and rural trends in average life-time parity, or total fertility of cohorts (TF), by country. We used cohort parity-progression ratios (PPR) which measure the proportion of women who have already had a given number of children and who go on to have an additional one. The TF is then estimated as a weighted average of the parities attained, with the weights being the parity distribution of women as implied by the chaining of the progression ratios from nulliparous to the first birth (PPR1) up to the progression from the fifth to the sixth birth (PPR6; see S1 File).

Survey-specific series of PPRs were computed for five-year age cohorts in order to increase robustness of the results. There is no missing information about parity in the data (while the fertility surveys do not allow missing data for this crucial question, statistical offices may have imputed values in the census records). For the cohorts that have completed their childbearing career at the time of the interview (i.e., aged 40 years or more), we directly estimated PPRs based on the distribution of women according to the reported parity at the survey date. Figures for cohorts with fewer than 60 women (in the unweighted sample) were discarded to avoid excessive noise in the estimation series due to sampling biases. In order to fill inter-cohort estimation gaps and extend the series with more recent cohorts, we estimated truncated PPRs of women aged 30–34 and 35–39 at the survey dates, and projected their completed PPRs.

We applied the Brass-Juarez paired-cohort comparison procedure to project these PPRs [28, 51]. The completed PPRs of older cohorts are adjusted for the cohort trend in fertility, which is estimated by accounting for the truncation of the fertility career and the selection of more fertile women in higher parity groups among younger cohorts (see S1 File). The method assumes that the recent fertility differentials between two adjacent cohorts stay constant in the second half of the reproductive career: no differential postponement or recuperation of births is allowed. Cross-validations of the Brass-Juarez approach with other indicators of parity progression have shown that the lengthening of birth intervals tends to significantly overestimate the fertility decline only among women aged less than 30 at the survey date [51]. We discarded these women in the present analysis. Survey weights were applied.

An internal and external quality assessment of our results confirms (1) the good quality of our data at older ages (i.e. the correct reporting of parity and the limited mortality selection of women) and (2) the negligible impacts of the assumptions used to project completed parity progression ratios based on truncated estimates for younger women (see S1 File). We thus averaged the PPRs obtained from successive surveys for overlapping cohorts, annually and linearly interpolated the figures and smoothed the country-specific series by type of residence (using the locally weighted least squares technique–the Stata Lowess function–with a bandwidth of 0.75 in all countries). Smoothing is important mainly for the most problematic series of PPRs from the fifth to the sixth birth in urban areas (see S1 File).

Urban and rural fertility trends were analyzed according to cohort years relative to the onset of the national transition. We first defined the last calendar year in which the national TFR peaked before the first 10%-decline–according to the United Nations' World Population Prospects estimations [2] and the compiled estimates for periods before 1950 [52]. We then back-translated the date of onset by subtraction of the period mean age at childbearing [2] in order to get a cohort indicator. This non-conventional definition of the transition onset (e.g. when fertility peaked) has the advantages of covering the early–and predominantly urban–fertility decline and better accounting for the slower trend in SSA, thereby accentuating regional differences in the speed of the transitions [22].

To estimate regional fertility trends, we first calculated the pace of change in urban and rural areas over cohorts, using linear spline regression models (with knots at the onset of the national transition and 10, 20, 30 and 40 cohorts later; we only considered those cohorts with observations for at least four countries). Country fixed effects control for the unobserved heterogeneity in the national contexts of urbanization and in urban/rural definitions. The regression coefficients were then used to predict regional average within-country trends. Country-specific results illustrate the variations in the fertility developments within regions and illuminate atypical trends, but the differences are not directly interpretable due to the lack of comparability of the rural/urban definitions.

## Results

### Regional variation in the evolution of the rural-urban fertility ratio

**Fig 2:**
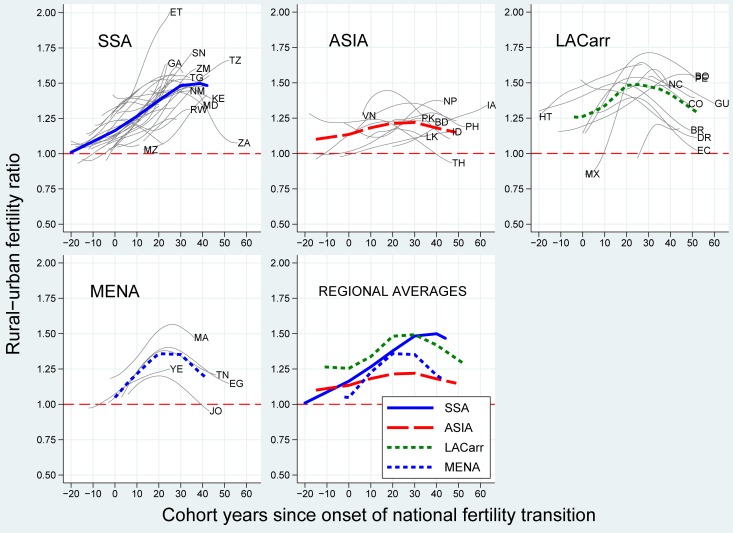
Average and country-specific trends in the rural-urban cohort fertility ratio over the course of the national fertility transitions (onset = year 0), cohorts 1896–1982 in 60 African, Asian and Latin American countries. Source: WFS & DHS, MICS, IPUMS, UN historical and World Population Prospects estimates (2017). Notes: thick lines designate average within-country trends. LACarr = Latin America and the Caribbean, MENA = Middle East and Northern Africa, SSA = sub-Saharan Africa; see Table 1 in S1 File for country acronyms; the onset of the transition corresponds to the calendar year in which the national-level TFR peaked, which was then back-translated by the mean age at birth to get a cohort indicator.

Fig 2 shows the trend in the rural-urban ratio of cohort fertility over the course of the national fertility transitions by developing region (thick lines) and country (thin lines).

No trend aligns on the red and dashed horizontal line at unity, meaning that urban and rural fertility did not decline at the same time and same pace. The majority of estimates are situated above this reference line, indicating a higher fertility in rural relative to urban subgroups of cohorts. The results tend to support the hypothesis of an inverted U-shaped trend in the rural-urban fertility ratio over time in all world regions, except in SSA.

However, the form of this U-shape differed across regions. Among the first transition cohorts in LACarr, rural fertility was 25% higher than the urban level on average. Rural excess fertility increased sharply in the first twenty transition cohorts (to a ratio of 1.5 on average), and flattened out later on. The average rural-urban ratio started to drop after 30 transition cohorts. In the most recent cohorts, the ratio remained above 1.25. Our data allow us to observe a significant decline in rural excess fertility in eight out of the 12 countries in the region. In the MENA, the average trend was generally similar. Three out of the four countries in the sample revealed a decline of the rural-urban fertility ratio. A residual gap also persists in the youngest cohorts.

The Asian region stands out with the lowest overall level of rural excess fertility. The inverted U-shaped trend over the cohorts is the flattest. Starting from an average ratio of 1.15 at the transition onset, it increased to slightly below 1.25 and declined thereafter. This drop is observed in four out of the 10 countries covered in the region.

In SSA, the rural-urban fertility ratio increased monotonically over the course of the national transition: from unity among cohorts born 10 years before the transition onset to 1.5 fifty cohorts later. Country-specific levels of rural excess fertility peaked at up to twice the urban level. Although there is some evidence for a trend reversal after the 40^th^ transition cohort, the SSA rural fertility decline is not advanced enough to make a definitive statement (see S1 File). A significant decrease in the rural-urban fertility ratio is observed in only two out of the 31 sub-Saharan African countries in the sample.

In sum, the extent of the rise in the rural-urban fertility ratio and the timing of its peak differed by world region. The role played by the underlying fertility dynamics by place of residence is analyzed in the following sections.

### The universal urban fertility decline

**Fig 3:**
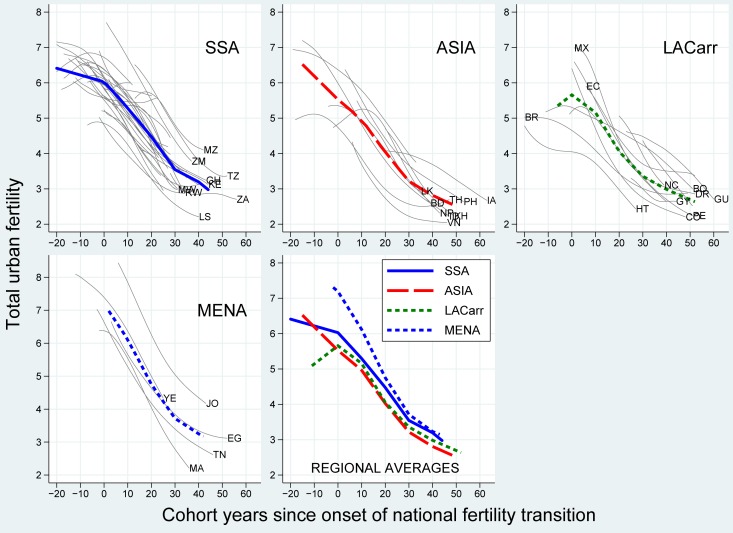
Average and country-specific trends in urban cohort fertility over the course of the national fertility transitions (onset = year 0), cohorts 1896–1982 in 60 African, Asian and Latin American countries. Source: WFS & DHS, MICS, IPUMS, UN historical and World Population Prospects estimates (2017). Notes: thick lines designate average within-country trends; LACarr = Latin America and the Caribbean, MENA = Middle East and Northern Africa, SSA = sub-Saharan Africa; see Table 1 in S1 File for country acronyms; the onset of the transition corresponds to the calendar year in which the national-level TFR peaked, which was then back-translated by the mean age at birth to get a cohort indicator.

Fig 3 shows the trends in urban fertility over the stages of the national fertility transitions by world region (thick lines) and country (thin lines). Regional variations would explain the differences in the steepness of the rise in the rural-urban fertility ratio before rural fertility starts to fall.

Yet the average trends in urban fertility were similar across world regions. At the national transition onset in LACarr, the MENA, and Asia, urban fertility was already declining sharply or slightly below 6 children per woman. The pre-transitional rise in urban fertility observed in many countries can be associated with the diffusion of public health and the renunciation of traditional practices of birth regulation [53]. Average urban fertility fell from 6 to 3 children per woman over the first 40 transition cohorts in all world regions, and reached 2.5 after 50 years in Asia and LACarr. These declines were fast and continuous. In LACarr, the average trend started to level off among the cohorts born 20 years after the transition onset. In Asia and the MENA, the monotonic decline in fertility lasted ten cohorts longer, but also slowed down later on.

This strong regional similarity in the urban fertility decline implies that the rural-urban fertility ratio could have increased strongly in all world regions. Yet it did not. The diversity between regions must be related to the fertility trends in rural areas.

### Regional differences in the urban-rural lags in the transition onset

The regional differences in the early rise and peak of the rural-urban fertility ratio could be attributed to the varying time lags between the urban and rural onsets of the fertility transition. This would confirm theories of the spatial diffusion of structural and ideational change. Table 1 shows for each world region the quartile years elapsed between the urban and rural onsets of advanced fertility decline in each country. These sector-specific transition onsets are defined by the first (urban/rural) cohort year in which total fertility dropped below the threshold of six children per woman (or the cohort with the highest fertility in the few urban areas where the level was never above six). We used survival analysis to estimate the timing of the rural, relative to the urban, onset in order to consider both the closed and the truncated intervals as observed in 50 countries. We excluded 10 countries where our data do not cover the early stage of the fertility transition (i.e. series starting after the tenth cohort year following the national transition onset): Cambodia, Guatemala, Guyana, Honduras, India, Mozambique, Nicaragua, Swaziland, Tanzania, and South Africa.

**Table 1:** Median and interquartile range of the years elapsed between the urban and rural onsets of advanced fertility decline, cohorts 1896–1982 in 50 African, Asian and Latin American countries.

World Region	Median lag	Variation (interquartile range)	Number of countries
ASIA	5	8	8
MENA	13	7	5
LACarr	16	12	10
SSA	21	14	27

Source: WHS, DHS, MICS, IPUMS.

Note: LACarr = Latin America and the Caribbean, MENA = Middle East and Northern Africa, SSA = sub-Saharan Africa; the onset of advanced fertility decline in each country and type of residence location is defined by the first transition cohort with a fertility level below six children per woman; 10 countries are not considered here due to the left-truncated series of urban fertility (i.e. starting after the tenth cohort year following the national transition onset; see country list in note 1).

The shortest median lag between the sectoral transition onsets is found in Asia (only 5 years). This explains its comparatively low peak in the rural-urban fertility ratio. The MENA follow behind with a 13 years lag. LACarr and SSA are characterized by the longest intervals, of 16 and 21 years respectively. In addition, LACarr and SSA reveal large interquartile ranges in the urban-rural transition lags. In SSA, the intercountry variation of 14 years represents approximately twice the variation observed within Asia and within the MENA. In eight SSA countries, rural areas have in fact not yet experienced advanced fertility decline. These long median lags between the urban and rural transition onsets account for the higher peak level of the rural-urban fertility ratio in LACarr and for its continuous increase in SSA.

### Urban-rural differences in the paces of fertility decline

**Fig 4:**
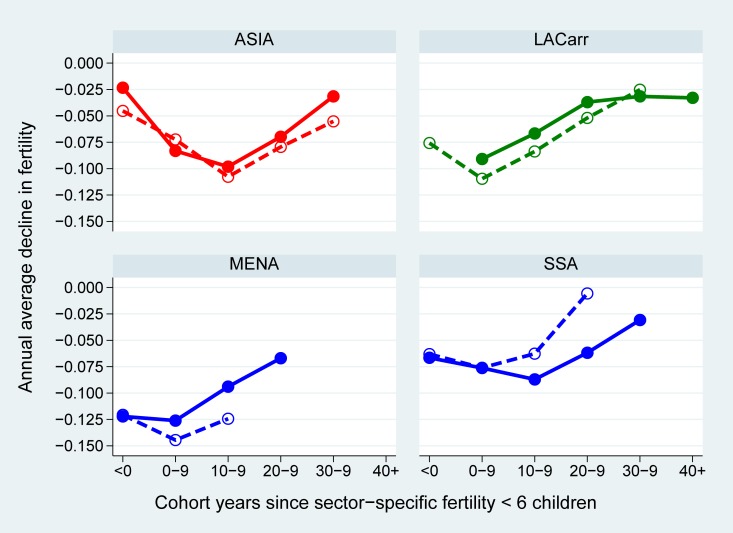
Regional averages of the annual pace of cohort fertility decline in urban and rural areas (full and dashed line, respectively) over the course of the national and sector-specific fertility transitions, cohorts 1896–1982 in 50 African, Asian and Latin American countries. Source: WFS & DHS, MICS, IPUMS. Notes: full lines indicate the urban pace, dashed lines indicate the rural pace; LACarr = Latin America and the Caribbean, MENA = Middle East and Northern Africa, SSA = sub-Saharan Africa; the onset of the sector-specific transition corresponds to the first cohort with total fertility below 6 children per woman.

Once rural fertility has started to decline, the rural-urban fertility ratio is determined by the differential paces of the transition by type of residence location. To evaluate rural-urban differences in the pace of fertility decline by world region, we plotted in Fig 4 the average annual rates of fertility change after the country- and sector-specific onset of advanced fertility decline (as defined in the previous sub-section).

In general, the average urban and rural pace of fertility decline follows a similar U-shaped trend in all world regions once it has started: from an annual decline of about -0.075 children per woman in the first (two) transition decades, the pace tends to slow down to about -0.025 in the fourth decade. Once the rural transition had started, therefore, the rural pace of decline was significantly faster when compared to that observed in the urban areas, which was already more advanced. This explains the drop in the rural-urban fertility ratio from its peak level in all regions, except in SSA (see below). Moreover, the fertility decline slowed down earlier over the course of the urban when compared to the rural fertility transition in Asia, LACarr and the MENA. This intensified the shrinkage of the rural-urban fertility ratio after its peak.

Unlike the other regions, the pace of fertility decline in SSA decelerated more rapidly in the countryside when compared to the cities. This implies that, even though fertility eventually started to drop in a number of rural areas, the slow rhythm (relative to the dynamics in urban areas) sustained the continuous increase in the rural-urban fertility ratio, instead of significantly inverting the trend.

### The role of rural-to-urban migration

**Fig 5:**
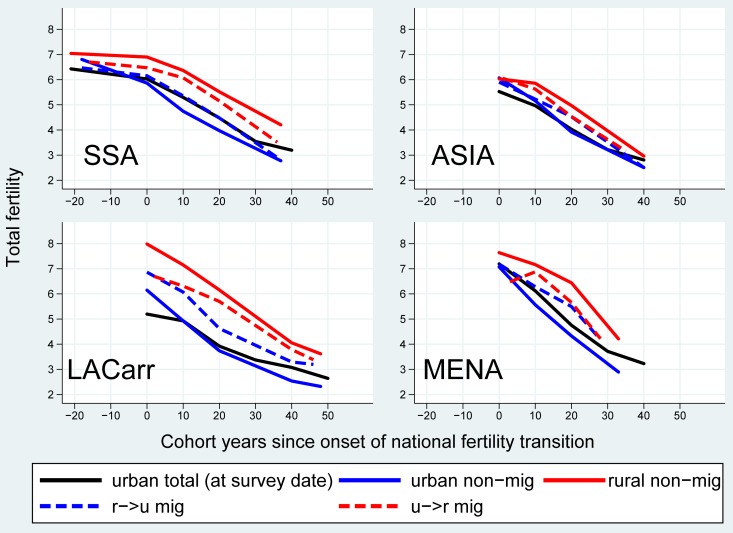
Regional average trend in cohort fertility among migrant and non-migrant women in urban and rural areas over the course of the national fertility transitions (onset = year 0), cohorts 1925–1978 in 55 African, Asian and Latin American countries. Source: WFS & DHS with information on migration and urban/rural status of the previous place of residence. Notes: LACarr = Latin America and the Caribbean, MENA = Middle East and Northern Africa, SSA = sub-Saharan Africa; the onset of the transition corresponds to the calendar year in which the national-level TFR peaked, which was then back-translated by the mean age at birth to get a cohort indicator.

The analysis above is confounded by the demographic effect of rural-to-urban migrants, who are included in the urban estimates. Not only have these migrants been socialized to higher fertility norms in the countryside, but the process of adaptation to the lower urban fertility patterns is generally completed only among their descendants, who have been socialized in cities [54–56]. Therefore, urban in-migrants may inflate urban fertility when compared to the trend among long-term urban dwellers. To appreciate whether this accounts for the regional difference in the evolutions in the rural-urban fertility ratio, we disaggregate the analysis further by migrant status (Fig 5). We expect in-migrants to be characterized by fertility levels which are intermediary between the levels of the rural and the urban non-migrants, which would decelerate the urban fertility decline.

Migrants are defined as women who have been socialized (in childhood) in another type of residence location when compared to that in which they have been interviewed, using 146 DHS and WFS with information on migration covering 55 countries (see S1 File). The fertility trend among non-migrants in urban areas is also compared with that among the total urban population at the survey-date in order to assess the impact of the in-migrants’ fertility.

In all world regions, rural-to-urban migrants constitute the large majority of movers. Their fertility levels were in between those of the non-migrants in the rural and the urban areas. However, the importance of the indirect effect of migration on urban fertility varies by world region, as it depends on both the difference in non-migrant versus migrant fertility and the level of urbanization.

In LACarr, the fertility differential between urban in- and non-migrants was the largest and remained constant over cohorts (0.8 births on average). The in-migrants’ impact on urban fertility levels, however, was negligible in the first thirty transition cohorts because the level of urbanization in Latin America was already relatively high (i.e. in-migrants represented a smaller proportion of the destination populations, when compared to other developing regions). Migrants started to inflate urban fertility only in the last transition stage (by up to 0.5 births) when the non-migrants’ fertility reached near replacement levels. In the MENA, the patterns were similar. The migrants’ impact in inflating urban fertility levels was more sustained and started earlier on in the transition because the level of urbanization was lower.

In SSA, the fertility differential between rural-to-urban migrants and urban non-migrants was comparatively more limited (0.3 births on average). Yet the inflation of urban fertility due to in-migration started immediately after the transition onset because the city populations were small (i.e. the levels of urbanization were much lower than in other regions). Migrants were responsible for at least 0.4 births more per woman in the total relative to the non-migrant population in urban areas. The Asian region is characterized by the smallest differentials in the levels of childbearing by migrant status (0.2 births on average), which exerted negligible effects on the trend in urban fertility at advanced stages of the transition (inflating the level by 0.3 births at best).

In sum, the indirect demographic effect of in-migrants (through their higher fertility) decelerated the urban fertility decline in the early stages of the transition in less urbanized regions (most notably in SSA), and in later stages of the fertility transition in all regions. Therefore, migration at least partially explains why the late urban fertility decline decelerated to a larger extent when compared to the rural trend, pushing the rural-urban fertility ratio closer towards unity. In SSA, the early and significant deceleration of the urban fertility decline caused by the arrival of in-migrants also accounts for the slower pace of the initial increase in the average rural-urban fertility ratio.

## Discussion and conclusion

Regional differences in the pace of urban fertility decline and the lagged dynamics in rural areas of developing countries are crucial for better understanding demographic change in an increasingly urbanized world. We examined whether the inverted U-shaped evolution in the rural-urban fertility ratio is confirmed from a longitudinal and long-term perspective of four developing regions, and we investigated the differences in the underlying dynamics by type of residence.

The results corroborated an increase followed by a decrease in rural excess fertility over the course of the fertility transition in all developing regions. At the same time, the rise and peak level of this excess fertility, as well as its later decline, differed. We found a marked inverted U-shaped evolution in the rural-urban fertility gradient only in LACarr and the MENA. Rural excess fertility rose continuously over an extended number of cohorts in SSA and has not yet significantly decreased. In Asia, the rural-urban fertility ratio remained comparatively the smallest. This regional variation was not due to differential paces in the urban trends. Our results reveal a fast and continuous urban fertility decline once it has started in all developing regions, including SSA which is often believed to follow an exceptional pathway. This confirms the importance of the universal process of fast structural and ideational change in societies for the transformation of reproductive behaviors.

Regional variations in the evolution of rural excess fertility are mainly attributable to the time lag until the fertility transition starts in rural areas. This confirms the major importance of the spatial diffusion of structural and ideational changes for national fertility decline. In Asia, the overall limited rural-urban gradient can be related to the fastest inter-sectoral diffusion of the onset of the fertility transition. By contrast, the rural transition onset lagged significantly behind the urban fertility decline in the MENA, LACarr and, especially, in SSA. This explains the stronger increase in rural excess fertility in these regions. In addition, the speed of fertility decline was less pronounced in rural than in urban areas of SSA–as opposed to other developing regions in which the two lagged transitions evolved at a similar pace. This peculiarity explains why rural excess fertility has not yet declined in SSA. The regional comparison is robust as we controlled for international variation in the demographic context and for intra-regional differences in the definition of urban/rural areas. Although we did not account for the inter-regional variations in the definitions, this is unlikely to challenge the main conclusions of this study. The rural-urban fertility gradient was limited in Asia, where urban environments are best identified. By contrast, the gradient in LACarr and, especially, SSA may be underestimated because officially designated urban areas tend to include agricultural communities.

The descriptive approach of this study does not allow us to identify explanations for the atypical fertility trends by type of residence in SSA and Asia. But our contextual knowledge enables us to speculate about potential causes. Even though the low level of urbanization in Asia would imply a slower urban-to-rural diffusion of behavioral change, authoritarian family planning policies are probably key to understanding the near parallel decline in fertility by type of residence. Alternatively, the limited rural-urban fertility gradients may indicate a loose relationship between the transformations in the socioeconomic structures of urban and rural populations and their respective levels of fertility, especially in the formerly communist countries of the region. While the fertility transition was fast, the urbanization process was restrained by communist governments [57]. Future research is needed to better understand the socioeconomic correlates of the fast rural fertility decline in this world region.

The reasons for the late and slow diffusion of fertility decline from SSA’s cities to the countryside remain an open question with important demographic consequences. The region clearly cumulates structural characteristics that potentially impede the spread of fertility change across space: a low level of urbanization, a limited integration of the agricultural sector in the national development process and a weak commitment of governments to improve the provision of family planning in remote areas. Hence the rural fertility transition appears to be disconnected from that in urban areas. Even though education expanded in rural areas, its impact on fertility decline was also less important when compared to urban areas [19]. Future research on the correlates of the urban-to-rural diffusion of the fertility transition in the region should account for the interactions between the two subnational population strata, including the potential role of rural-to-urban migration in the conservation of traditional livelihood strategies in the countryside.

The results of this study help to better understand the variations in the fertility transitions across developing regions. The fast fertility decline in LACarr and the MENA can be related, first, to the dominance of cities in the population geography when urban fertility started to fall. Second, the minority population remaining in rural areas caught up the decline at a similarly fast pace. Given the high level of urbanization in these regions, future national-level trends will be mainly determined by the fertility dynamics in urban areas. By contrast, the synchronized decline in urban and rural fertility in Asia explains why this region experienced the fastest fertility transition in worldwide comparison–despite a low level of urbanization. Given a persistent residual level of rural excess fertility, further progress in urbanization will significantly shape prospective fertility trends in the region.

In SSA, the national-level trends of slow fertility decline in conjunction with rampant socio-economic development [21, 22] actually concerns only the rural areas of the region. These dominated the national fertility transitions because the level of urbanization was low and progressed at a slower pace than elsewhere. Our results also amend the conventional wisdom which explains SSA’s exceptional transition as being mainly driven by birth spacing [28, 29]. The fast decline in urban fertility suggests that stopping behaviors of childbearing emerged early on in cities, although this proposition needs to be verified by further research. Future fertility developments in this region will rest on both the acceleration of the decline in rural fertility and the continuing progress of urbanization. The lessons from the marketing campaigns for modern contraception in rural areas of LACarr and MENA can inform policies that aim to speed up the SSA fertility transition. Additional efforts should be invested in strengthening family planning programs and ensuring equal access in remote areas.

The results also have implications for the future geography of urban growth. Given that fertility has declined continuously among women socialized in cities, migration eventually dominates the urban growth process through both its direct and its indirect demographic contributions. Urban growth can therefore be expected to be concentrated in Asia and SSA, where the pool of potential in-migrants remains large due to the low levels of urbanization. In SSA, the future urban-to-rural diffusion of fertility decline will be crucial to limiting demographic pressure for rural-to-urban migration and, consequently, the acute strains on urban infrastructure and labour markets, as well as the resulting pressure for secondary migrations to foreign countries.

## Supporting information

S1 File Appendix: Data, method, and cross-validation of estimates. (DOCX)

S2 File Fertility estimates by country, type of place of residence and cohort. (DTA)
