# Supplementary material for: Regional variations in the rural-urban fertility gradient in the global South
Source: PLoS One. 2019 Jul 19;14(7):e0219624. doi: 10.1371/journal.pone.0219624 (PMC6641161; doi:10.1371/journal.pone.0219624)
Supplement: S1 File — (DOCX) [file pone.0219624.s001.docx]

# Appendix: data, method, and cross-validation of estimates

## Data

The survey and census data are representative at the national and urban-rural level. Table 1 lists the sample of countries by world region, alongside the survey waves and census rounds used, the cohorts covered by the data, as well as the first and last cohort fertility estimates by type of residence location. In the WFS, DHS and MICS, women have been selected for interview according to a classic stratified two-staged random sampling with replacement. The IPUMS samples contain between 5 and 10% of households randomly selected among the exhaustive population.

*S-Table 1: about here*

Our efforts to complement the WFS and DHS data with earlier and later household surveys or censuses in countries with a limited number of fertility surveys minimized to a large extent the analytical problems related to unbalanced panels of country series. Nevertheless, we do not cover the first ten transition cohorts in ten countries (i.e. Cambodia, Guatemala, Guyana, Honduras, India, Mozambique, Nicaragua, Swaziland, Tanzania, and South Africa). The series are also truncated before the 30^th^ transition cohort in Haiti, Yemen and 15 sub-Saharan countries for reasons not related to the outcome under study: the fertility transition started later and is still unfolding.

## Method

To project completed PPRs of younger cohorts, the Brass-Juarez paired-cohort comparison procedure was applied [1, 2]. The truncated PPRs for the younger cohorts are projected forward in time by multiplying the completed PPR of the cohort aged 40-44 at the survey date *t* (*PPR(40-44, t)* in the Equation below) by that cohort’s fertility differential with the immediately younger cohort and by the latter cohort’s fertility differential with the subsequent cohort (second right-hand term in the Equation below). In other words, the completed PPRs are multiplied with the downward-cumulated fertility change ratios between successive pairs of adjacent cohorts.

$$PPR\left( 30-34, t+10 \right)=PPR\left( 40-44,t \right)* \prod_{c=35-39}^{40-44} {\frac{PPR(c-5,t)}{PPR(c,t-5)}}$$

The innovative idea of the Brass-Juarez method is to estimate the inter-cohort fertility change ratios at equivalent ages and parities in order to control for the truncation of the fertility career and the selection of more fertile women in higher parity groups among younger cohorts. For the older women in each pair of cohorts, the number of births that occurred in the five-year period immediately preceding the survey (as reported in the birth histories) are subtracted from the stated parity at the survey date *t*. Thus, the older cohort’s PPR as of five years before the survey (*PPR(c, t-5)*) is truncated and affected by selection to the same extend as the younger cohort’s PPR at the time of the survey (*PPR(c-5, t)*).

The level of TF is then estimated as a weighted average of the parities attained, with the weights being the parity distribution of women as implied by the chaining of the progression ratios from nulliparous to the first birth (*PPR0->1*) through the ratio of progression from the fifth to the sixth birth (*PPR5->6*):

$$TCF=\left[ \sum_{i=1}^{5} i*{PPR}_{0\to1}*\ldots*\left( 1-{PPR}_{i\to i+1} \right) \right]+avCEBP6*{PPR}_{0\to1}*\ldots* {PPR}_{5\to6}$$

For the last parity group (women with at least six births), the attained parity was estimated as the average number of children ever born to this group at the survey dates (*avCEBP6*); inter-survey figures are linearly interpolated, the estimates before the first survey are extrapolated, and the series have been smoothed.

## Cross-validating the estimates

The information about the number of children ever born may be biased due to the older interviewee’s omission or incomplete recalling of births, as well as sample selection biases related to past mortality and migration [3, 4]. We performed two quality tests of our estimations series. The first is an internal plausibility test based only on the survey/census-samples. To assess the quality of the reporting of women’s parity, on the one hand, and the accuracy of our projections for younger cohorts, on the other hand, we cross-validated the observed and projected values of completed PPRs for overlapping cohorts as obtained respectively from two successive surveys. According to this internal plausibility test of our data, we found a higher agreement between observed and projected PPRs in rural areas and at lower parities, which can be explained by larger samples of women. S-Figure 1 shows the most problematic crude series of observed and projected progression ratios as obtained from successive surveys: the transition to the sixth birth (PPR6) in urban areas. Countries are purposively selected to illustrate the range of data quality. On each individual blue line, the last two points designate projected values, whereas prior points represent estimates. The averaged and smoothed trend is also plotted in red.

*S-Figure 1: about here*

In urban Bangladesh, the Philippines, Indonesia and Peru, the series almost perfectly overlap. Yet recent projection points from the penultimate surveys tend to be higher when compared with figures based on both the preceding and following surveys. This may have arisen because of different sample compositions. The examples of urban Togo, Vietnam and Kenya illustrate the worst cases in terms of the disagreement between estimated and projected PPRs. In Vietnam, the projections based on the penultimate survey are lower when compared with the estimates from the last survey. Younger birth cohorts may have shortened birth intervals (relative to older cohorts) at younger ages, but were not more likely to progress to the sixth birth (as assumed by the chaining of the inter-cohort fertility change ratios in the Brass-Juarez method). In urban Togo, the projected PPR6 from the first survey are significantly lower when compared to the estimated value based on the second survey. This points to a sample selection bias of more fertile women at the second survey. In urban Kenya, several biases may interact to produce the most erratic pattern observed in our sample of countries. Sample biases may be particularly important. In the 1998 DHS, for example, interviewers had no access to the households located within the slums which were protected by paramilitary groups (personal communication by Ph. Bocquier).

The estimates and projections of completed PPRs are generally the least congruent in the poorest countries within each world region. In these cases, not only the parity data may be of poorer quality, but also the forecast accuracy may be lower because of the underreporting of recent births in the birth history [5]. This leads to a miss-estimation of the inter-cohort fertility change ratios (used to project forward the truncated PPRs). Alternatively, the survey samples may not well represent the population due to sampling, migration and mortality-related selection biases. Changes in the urban classification between two surveys may also confound the comparison.

Overall, however, we can conclude that the quality of our series of completed PPRs is good. We thus smoothed the trend by, first, averaging data points for overlapping cohorts, linearly interpolating estimates and, then, applying a running line function (using the locally weighted least squares technique; see thick lines in A-Fig. 1). Survey weights are applied. We chained these smoothed PPRs to estimate the parity distribution of cohorts, which constitutes the weights to estimate the total cohort fertility (TF).

As a second external plausibility test, we estimated national-level total cohort fertility based on the PPRs (which are primarily based on parity data) and compared the trends with two external estimates: the United Nations’ period TFR series which have been back-translated by the average age at childbearing to get a cohort indicator, and Sneeringer’s [6] estimates of the total cohort fertility rates (CTFR; i.e. the sum of age-specific rates) based on the pooled birth histories from successive DHS in Africa. As shown in S-Fig 2, our estimates fit the two other series well – even in countries where only ever-married women have been interviewed (such as in Bangladesh, Egypt, Indonesia, and Morocco). The levels of cohort fertility diverge in Yemen and Haiti, but the trends are congruent. For some African countries, however, our parity-based estimates tend to indicate an earlier fertility decline when compared to the UN series. The CTFR based on the birth histories is situated either between our parity-based and the UN estimates, or are closer to our figures. In Chad, Burkina Faso, and Niger, however, we tend to under-estimate fertility among the most recent cohorts.

Rural cohort fertility trends over the course of the national fertility transitions are given in S-Fig 3 for comparison purposes with Fig. 2 in the main body of this manuscript.

*S-Figure 3: about here*

## Identification of migrants between urban and rural areas

To identify this population migrants, we relied upon the information about the urban/rural status of the childhood place of residence (sometimes defined until age 12) as reported in the WFS and some DHS, and about the status of the previous place of residence (alongside the date of the last move) which is available in several DHS (see Appendix). Although this information is self-reported and, therefore, susceptible to be biased, it reflects women’s perception of the environment of socialization, which is relevant for analyzing the diffusion of cohort fertility change. We assumed that women moved only once (at most) since childhood and harmonized the information with that provided in the WFS by identifying (non-)migrant women who did (not) move across urban/rural borders after age 15. The percentage of migrants was 22% of the interviewed women aged 30 or more in the average country, with a first percentile of 10% and a ninth percentile of 34%.

## Appendix Tables and Figures

*S-Table 1: Country-specific levels of urbanization, number and dates of fertility surveys, and urban and rural estimates of cohort fertility in 60 African, Asian and Latin American countries.*

|  | Country | | % urban | | Survey (census) years | | | | | | | | | | | | Obs. cohorts urban | | | | Obs. cohorts rural | | | |
| --- | --- | --- | --- | --- | --- | --- | --- | --- | --- | --- | --- | --- | --- | --- | --- | --- | --- | --- | --- | --- | --- | --- | --- | --- |
|  | (abbr. & name) | | 1950 | 2000 | 1st | 2nd | 3rd | 4th | 5th | 6th | 7th | 8th | 9th | 10th | 11th | 12th | First | TF | Last | TF | First | TF | Last | TF |
| ASIA | BD | Bangladesh | 4 | 24 | 1975 | 1993 | 1996 | 1999 | 2000 | 2004 | 2007 | 2011 | 2014 |  |  |  | 1926 | 7.2 | 1980 | 2.6 | 1926 | 6.9 | 1980 | 3.2 |
|  | KH | Cambodia | 10 | 19 | 2000 | 2005 | 2010 | 2014 |  |  |  |  |  |  |  |  | 1951 | 4.7 | 1980 | 2.2 | 1951 | 5.8 | 1980 | 2.8 |
|  | IA | India | 17 | 28 | 1992 | 1999 | 2000 | 2005 |  |  |  |  |  |  |  |  | 1928 | 4.1 | 1971 | 2.7 | 1928 | 4.3 | 1971 | 3.6 |
|  | ID | Indonesia | 12 | 42 | 1976 | 1987 | 1991 | 1994 | 1997 | 2002 | 2007 | 2012 |  |  |  |  | 1927 | 5.3 | 1978 | 2.2 | 1927 | 5.2 | 1978 | 2.6 |
|  | NP | Nepal | 3 | 13 | 1976 | 1996 | 2001 | 2006 | 2011 |  |  |  |  |  |  |  | 1932 | 4.7 | 1977 | 2.3 | 1927 | 5.7 | 1977 | 3.2 |
|  | PK | Pakistan | 18 | 33 | 1975 | 1990 | 2006 | 2012 |  |  |  |  |  |  |  |  | 1926 | 6.9 | 1978 | 3.8 | 1926 | 7.0 | 1978 | 4.7 |
|  | PH | Philippines | 27 | 48 | 1978 | 1993 | 1998 | 2003 | 2008 | 2013 |  |  |  |  |  |  | 1929 | 6.4 | 1979 | 2.6 | 1929 | 7.4 | 1979 | 3.1 |
|  | LK | Sri Lanka | 15 | 18 | 1975 | 1987 |  |  |  |  |  |  |  |  |  |  | 1926 | 5.5 | 1953 | 2.9 | 1926 | 6.1 | 1953 | 3.3 |
|  | TH | Thailand | 16 | 31 | 1970 | 1980 | 1987 | 2000 | 2005 | 2012 |  |  |  |  |  |  | 1906 | 5.0 | 1971 | 2.7 | 1906 | 6.4 | 1971 | 2.5 |
|  | VN | Viet Nam | 12 | 24 | 1989 | 1997 | 2002 | 2005 | 2006 | 2009 | 2014 |  |  |  |  |  | 1940 | 4.3 | 1980 | 2.0 | 1940 | 5.5 | 1980 | 2.3 |
| LACarr | BO | Bolivia | 34 | 62 | 1976 | 1989 | 1993 | 1998 | 2003 | 2008 |  |  |  |  |  |  | 1912 | 5.2 | 1974 | 3.0 | 1912 | 6.0 | 1974 | 4.6 |
|  | BR | Brazil | 36 | 81 | 1970 | 1980 | 1986 | 1996 | 2000 | 2010 |  |  |  |  |  |  | 1906 | 5.0 | 1976 | 2.9 | 1906 | 6.5 | 1976 | 3.4 |
|  | CO | Colombia | 33 | 72 | 1976 | 1986 | 1990 | 1995 | 2000 | 2004 | 2009 |  |  |  |  |  | 1927 | 6.4 | 1975 | 2.2 | 1927 | 7.3 | 1975 | 3.0 |
|  | DR | Dominican Rep. | 24 | 62 | 1975 | 1986 | 1991 | 1996 | 1999 | 2002 | 2007 | 2013 |  |  |  |  | 1926 | 5.1 | 1979 | 2.9 | 1926 | 7.8 | 1979 | 3.2 |
|  | EC | Ecuador | 28 | 60 | 1979 | 1987 | 2001 | 2010 |  |  |  |  |  |  |  |  | 1930 | 5.9 | 1976 | 3.4 | 1930 | 7.6 | 1976 | 3.5 |
|  | GU | Guatemala | 25 | 45 | 1987 | 1995 | 1998 | 2014 |  |  |  |  |  |  |  |  | 1943 | 4.6 | 1980 | 2.7 | 1943 | 6.8 | 1980 | 3.7 |
|  | GY | Guyana | 28 | 29 | 2005 | 2009 |  |  |  |  |  |  |  |  |  |  | 1956 | 3.3 | 1975 | 2.6 | 1956 | 3.2 | 1975 | 3.1 |
|  | HT | Haiti | 12 | 36 | 1977 | 1994 | 2000 | 2005 | 2012 |  |  |  |  |  |  |  | 1928 | 5.2 | 1978 | 2.4 | 1928 | 6.2 | 1978 | 4.0 |
|  | HN | Honduras | 18 | 45 | 2005 | 2011 |  |  |  |  |  |  |  |  |  |  | 1956 | 4.4 | 1977 | 2.5 | 1956 | 6.7 | 1977 | 3.5 |
|  | MX | Mexico | 43 | 75 | 1976 | 1987 | 2015 |  |  |  |  |  |  |  |  |  | 1927 | 7.0 | 1966 | 2.6 | 1927 | 6.0 | 1966 | 3.9 |
|  | NC | Nicaragua | 35 | 55 | 1997 | 2001 |  |  |  |  |  |  |  |  |  |  | 1948 | 5.1 | 1967 | 3.1 | 1948 | 7.4 | 1967 | 4.6 |
|  | PA | Panama | 36 | 62 | 1970 | 1975 | 1980 | 2000 | 2010 |  |  |  |  |  |  |  | 1906 | 3.3 | 1976 | 3.4 | 1896 | 6.2 | 1976 | 4.0 |
|  | PY | Paraguay | 35 | 55 | 1962 | 1979 | 1990 | 1992 | 2002 |  |  |  |  |  |  |  | 1898 | 2.6 | 1968 | 3.5 | 1898 | 3.7 | 1968 | 4.2 |
|  | PE | Peru | 41 | 73 | 1977 | 1986 | 1991 | 1996 | 2000 | 2004 | 2007 | 2009-2012 | |  |  |  | 1928 | 6.6 | 1978 | 2.2 | 1928 | 7.5 | 1978 | 3.4 |
| MENA | EG | Egypt | 32 | 43 | 1980 | 1988 | 1992 | 1995 | 2000 | 2003 | 2005 | 2008 | 2014 |  |  |  | 1931 | 6.7 | 1980 | 3.1 | 1931 | 7.0 | 1980 | 3.6 |
|  | JO | Jordan | 37 | 80 | 1990 | 1997 | 2002 | 2007 | 2009 | 2012 |  |  |  |  |  |  | 1941 | 8.4 | 1978 | 4.2 | 1941 | 9.0 | 1978 | 4.0 |
|  | MA | Morocco | 26 | 53 | 1980 | 1987 | 1992 | 2003 |  |  |  |  |  |  |  |  | 1931 | 6.4 | 1969 | 2.2 | 1931 | 7.6 | 1969 | 3.3 |
|  | TN | Tunisia | 32 | 63 | 1978 | 1988 | 2012 |  |  |  |  |  |  |  |  |  | 1929 | 7.0 | 1978 | 2.6 | 1929 | 7.1 | 1978 | 3.2 |
|  | YE | Yemen | 6 | 26 | 1991 | 2013 |  |  |  |  |  |  |  |  |  |  | 1942 | 8.1 | 1979 | 4.3 | 1942 | 7.9 | 1979 | 5.4 |
| SSA | BJ | Benin | 5 | 38 | 1981 | 1996 | 2001 | 2006 | 2011 |  |  |  |  |  |  |  | 1932 | 5.8 | 1977 | 4.2 | 1932 | 6.1 | 1977 | 5.6 |
|  | BF | Burkina Faso | 4 | 18 | 1992 | 1998 | 2003 | 2010 | 2014 |  |  |  |  |  |  |  | 1948 | 6.6 | 1980 | 4.0 | 1943 | 7.6 | 1980 | 6.2 |
|  | BU | Burundi | 2 | 8 | 1987 | 2010 | 2012 |  |  |  |  |  |  |  |  |  | 1943 | 6.0 | 1978 | 5.6 | 1938 | 7.1 | 1978 | 5.9 |
|  | CM | Cameroon | 9 | 46 | 1991 | 1998 | 2004 | 2011 |  |  |  |  |  |  |  |  | 1942 | 6.1 | 1977 | 3.9 | 1942 | 6.3 | 1977 | 6.4 |
|  | TD | Chad | 5 | 22 | 1996 | 2004 | 2014 |  |  |  |  |  |  |  |  |  | 1947 | 6.3 | 1980 | 5.9 | 1947 | 6.9 | 1980 | 7.4 |
|  | CI | Côte d'Ivoire | 10 | 44 | 1980 | 1994 | 1998 | 2005 | 2011 |  |  |  |  |  |  |  | 1931 | 6.6 | 1977 | 3.5 | 1931 | 6.8 | 1977 | 5.2 |
|  | ET | Ethiopia | 5 | 15 | 2000 | 2005 | 2011 |  |  |  |  |  |  |  |  |  | 1951 | 5.7 | 1977 | 3.0 | 1951 | 7.4 | 1977 | 5.9 |
|  | GA | Gabon | 11 | 80 | 2000 | 2012 |  |  |  |  |  |  |  |  |  |  | 1951 | 6.1 | 1978 | 3.4 | 1951 | 6.3 | 1978 | 5.6 |
|  | GH | Ghana | 15 | 44 | 1979 | 1988 | 1993 | 1998 | 2003 | 2008 | 2014 |  |  |  |  |  | 1930 | 6.0 | 1980 | 3.2 | 1930 | 6.6 | 1980 | 5.0 |
|  | GN | Guinea | 7 | 31 | 1999 | 2005 | 2012 |  |  |  |  |  |  |  |  |  | 1950 | 6.6 | 1978 | 4.0 | 1950 | 6.8 | 1978 | 5.9 |
|  | KE | Kenya | 6 | 20 | 1977 | 1988 | 1993 | 1998 | 2003 | 2008 | 2014 | 2015 |  |  |  |  | 1928 | 6.5 | 1981 | 3.1 | 1928 | 7.8 | 1981 | 4.3 |
|  | LS | Lesotho | 2 | 20 | 1977 | 2004 | 2009 | 2014 |  |  |  |  |  |  |  |  | 1933 | 4.8 | 1980 | 2.2 | 1928 | 5.3 | 1980 | 3.3 |
|  | LB | Liberia | 13 | 44 | 1986 | 2006 | 2008 | 2011 | 2013 |  |  |  |  |  |  |  | 1937 | 6.7 | 1979 | 3.6 | 1937 | 6.4 | 1979 | 5.5 |
|  | MD | Madagascar | 8 | 27 | 1992 | 1997 | 2003 | 2008 | 2011 | 2013 | 2016 |  |  |  |  |  | 1943 | 5.9 | 1982 | 3.1 | 1943 | 7.6 | 1982 | 4.2 |
|  | MW | Malawi | 4 | 15 | 1992 | 2000 | 2004 | 2010 | 2012 | 2014 | 2015 |  |  |  |  |  | 1948 | 6.9 | 1981 | 3.0 | 1943 | 7.2 | 1981 | 4.7 |
|  | ML | Mali | 8 | 28 | 1987 | 1995 | 2001 | 2006 | 2012 | 2015 |  |  |  |  |  |  | 1938 | 7.3 | 1981 | 5.6 | 1938 | 7.0 | 1981 | 7.5 |
|  | MR | Mauritania | 3 | 49 | 1981 |  |  |  |  |  |  |  |  |  |  |  | 1932 | 6.3 | 1947 | 6.0 | 1932 | 6.0 | 1947 | 6.4 |
|  | MZ | Mozambique | 3 | 29 | 1997 | 2003 | 2011 |  |  |  |  |  |  |  |  |  | 1948 | 5.6 | 1977 | 4.1 | 1948 | 5.7 | 1977 | 5.8 |
|  | NM | Namibia | 13 | 32 | 1992 | 2000 | 2006 | 2013 |  |  |  |  |  |  |  |  | 1943 | 5.0 | 1979 | 2.9 | 1943 | 6.3 | 1979 | 4.2 |
|  | NI | Niger | 5 | 16 | 1992 | 1998 | 2006 | 2012 |  |  |  |  |  |  |  |  | 1943 | 7.3 | 1978 | 5.0 | 1943 | 7.8 | 1978 | 7.2 |
|  | RW | Rwanda | 2 | 15 | 1992 | 2000 | 2005 | 2007 | 2010 | 2013 | 2014 |  |  |  |  |  | 1948 | 6.1 | 1980 | 2.9 | 1943 | 8.2 | 1980 | 3.8 |
|  | SN | Senegal | 17 | 40 | 1978 | 1986 | 1992 | 1997 | 1999 | 2005 | 2006 | 2008 | 2010 | 2012 | 2014 | 2015 | 1929 | 6.8 | 1981 | 3.3 | 1929 | 7.1 | 1981 | 5.7 |
|  | SL | Sierra Leone | 13 | 36 | 2008 | 2013 |  |  |  |  |  |  |  |  |  |  | 1959 | 5.2 | 1979 | 4.0 | 1959 | 5.8 | 1979 | 5.3 |
|  | ZA | South Africa | 42 | 57 | 1998 | 2007 | 2011 |  |  |  |  |  |  |  |  |  | 1949 | 3.4 | 1977 | 2.7 | 1949 | 5.0 | 1977 | 2.9 |
|  | SD | Sudan | 7 | 32 | 1978 | 1989 | 2008 |  |  |  |  |  |  |  |  |  | 1929 | 6.1 | 1974 | 4.5 | 1929 | 5.7 | 1974 | 5.5 |
|  | SZ | Swaziland | 2 | 23 | 2006 |  |  |  |  |  |  |  |  |  |  |  | 1957 | 4.1 | 1972 | 3.0 | 1957 | 5.9 | 1972 | 4.3 |
|  | TG | Togo | 4 | 33 | 1988 | 1998 | 2013 |  |  |  |  |  |  |  |  |  | 1939 | 6.1 | 1979 | 3.4 | 1939 | 7.7 | 1979 | 5.2 |
|  | UG | Uganda | 3 | 12 | 1988 | 1995 | 2000 | 2006 | 2009 | 2011 | 2014 |  |  |  |  |  | 1944 | 6.7 | 1980 | 4.3 | 1939 | 7.7 | 1980 | 6.5 |
|  | TZ | Tanzania | 3 | 22 | 1991 | 1996 | 1999 | 2004 | 2007 | 2009 | 2011 | 2015 |  |  |  |  | 1942 | 6.5 | 1981 | 3.4 | 1942 | 7.1 | 1981 | 5.6 |
|  | ZM | Zambia | 12 | 35 | 1992 | 1996 | 2001 | 2007 | 2013 |  |  |  |  |  |  |  | 1943 | 7.7 | 1979 | 3.8 | 1943 | 8.1 | 1979 | 6.1 |
|  | ZW | Zimbabwe | 11 | 34 | 1988 | 1994 | 1999 | 2005 | 2010 | 2015 |  |  |  |  |  |  | 1939 | 5.2 | 1981 | 3.2 | 1939 | 7.4 | 1981 | 4.7 |

Sources: WHS & DHS, MICS, IPUMS, UN 2015.

*Notes: LACarr = Latin America and the Caribbean, MENA = Middle East and Northern Africa, SSA = sub-Sahara Africa.*

*S-Figure 1: Survey-specific estimates and projections of transition ratios from the fifth to the sixth birth (in blue; PPR6) and the smoothed trend (in red) in urban areas, cohorts 1926-1982 in selected Asian, Latin American and African countries.*


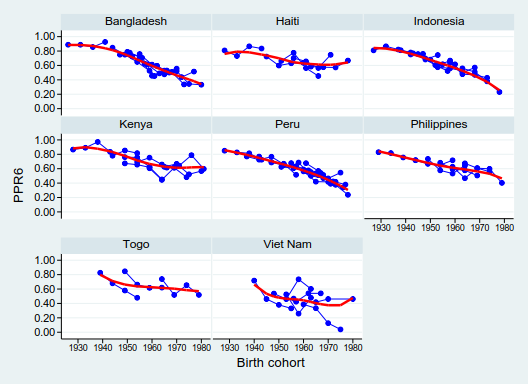


Source: DHS & WHS.

Note: on each individual blue line, the last two points designate projected values, whereas prior points represent estimates; the survey-specific estimates and projections of lower parity transitions in urban areas, as well as estimates for rural areas, are not shown because they are more congruent to each other when compared to the urban estimates of the transition from the fifth to the sixth birth*.*

*S-Figure 2: Three estimates of the national-level total cohort fertility trends, as implied by the chaining of cohort PPRs, by cohort age-specific fertility rates, and by the back-translation of period TFRs (by the mean age at birth), cohorts 1896-1982 in selected Asian, Latin American and African countries.*


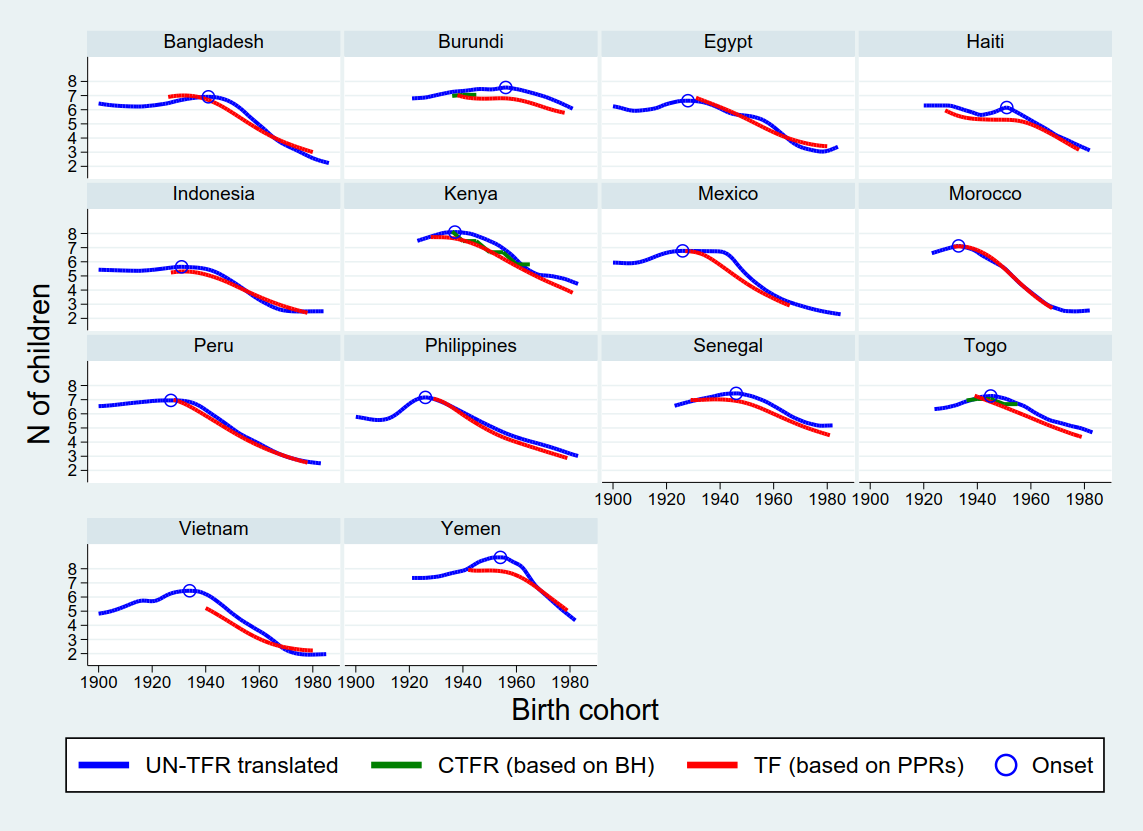


Sources: WFS & DHS, UN World Population Prospects 2015, Sneeringer (2009). Note: BH = Birth history, PPR = parity progression ratio.

*S-Figure 3: Average and country-specific trends in rural cohort fertility over the course of the national fertility transitions (onset = year 0), cohorts 1896-1982 in 60 African, Asian and Latin American countries.*


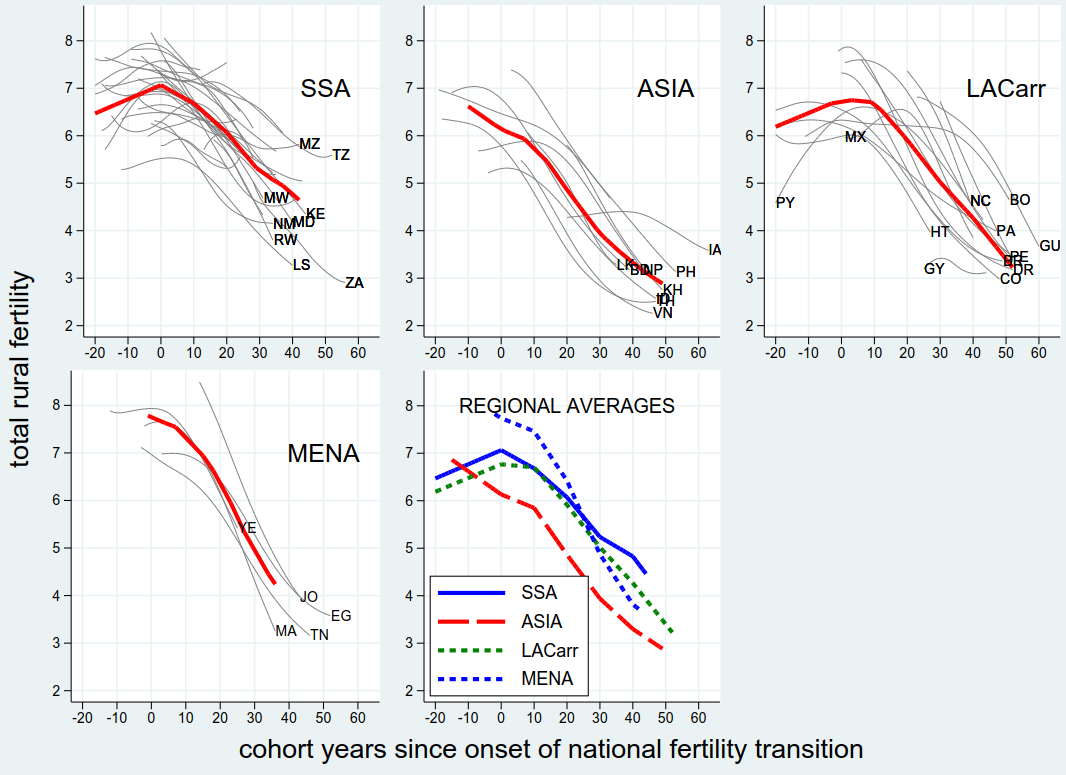


Source: WFS & DHS, MICS, IPUMS, UN historical estimates and World Population Prospects (2017).

Notes: thick lines designate average within-country trends; fine blue lines designate countries with long estimations series; LACarr = Latin America and the Caribbean, MENA = Middle East and Northern Africa, SSA = sub-Saharan Africa; see Table 2 in Appendix for country acronyms; the onset of the transition corresponds to the calendar year in which the TFR peaked, which was then back-translated by the mean age at birth to get a cohort indicator*.*

## References

1. Moultrie TA, Sayi TS, Timaeus IM. Birth intervals, postponement, and fertility decline in Africa: A new type of transition. Population Studies. 2012;66(3):241-58.

2. Brass W, Juarez F. Censored Cohort Parity Progression Ratios from Birth Histories. Asian and Pacific Census Forum. 1983;10(1):5-13.

3. Moultrie TA, Dorrington R, Hill A, Hill K, Timaeus I, Zaba B. Tools for Demographic Estimation. Paris: International Union for the Scientific Study of Population (IUSSP); 2013.

4. United Nations. Manual X: Indirect Techniques for Demographic Estimation. Affairs DoIEaS, editor. New York: United Nations; 1983.

5. Schoumaker B. Quality and Consistency of DHS Fertility Estimates, 1990 to 2012. DHS Methodological Reports. 2014;12:122.

6. Sneeringer SE. Fertility Transition in Sub-Saharan Africa: A Comparative Analysis of Cohort Trends in 30 Countries. Calverton, Maryland, USA: ICF Macro; 2009. Contract No.: 23.
